# Keratoconus: A Probe Into the Refractive Symmetry

**DOI:** 10.1155/joph/3827883

**Published:** 2025-10-15

**Authors:** Sankhajyoti Saha, Moubani Dutta, Soumendra Nath Bandyopadhyay, Pratyay Ranjan Dutta, Agnihiya Bosu

**Affiliations:** ^1^Department of Health Science, NSHM Knowledge Campus, Kolkata, West Bengal, India; ^2^Department of Computing and Analytics, NSHM Knowledge Campus, Kolkata, West Bengal, India; ^3^Department of Economics, National University, Gazipur, Bangladesh

**Keywords:** asphericity coefficient, corneal diopter, corneal thickness, keratoconus, mirror symmetry

## Abstract

**Background:**

A debilitating corneal ectasia, designated as keratoconus, often leads to distorted and obscured vision, and greater reactivity to light. Inevitably, the cornea becomes thinner and protrudes outward forming a cone-like configuration. The research hypothesis is initiated to estimate the reliability of the mirror symmetry and the dimensions of keratoconus severity, prospecting that mirror octant possessed a significant impact on the trajectory of the disorder.

**Methods:**

This study included patients with clinically diagnosed with bilateral keratoconus and bilateral astigmatism. Mirror symmetry or enantiomorphism was quantified employing the refractive cylindrical notations of yoke eye. Pentacam enable the observation of keratoconus severity utilizing corneal thickness, average corneal thinning, and asphericity coefficient. Multiple *R* was performed to analyze the model fit along with descriptive statistics. One-way ANOVA, guided by F-statistics, was solicited to analyze group variability, while a scatter plot was exploited to forecast the direction of mirror octant association of the variables. SPSS 29.0 software was utilized to perform the statistical analysis, with a *p* value of less than 0.05 was considered statistically significant.

**Result:**

With a significant (*p* < 0.05) F-statistic, the mirror symmetry remains the statistically significant predictor in the regression model. Average corneal diopter for both eyes exhibit a positive correlation. Conversely, corneal thickness and asphericity coefficient for both eyes demonstrate a negative correlation with mirror symmetry.

**Conclusions:**

Mirror symmetry's applicability may be inadequate by its subservience on corneal contour analysis, which, although obliging, may abstruse other salient clinical considerations. With 35.6% of the model variability, it indicates a room for improvement by adding nonlinear predictors to enhance the model.

## 1. Introduction

Biological layouts are necessary to sustain physiological functionality. One can identify conventional and impulsive oscillations in biomolecule ratios. To serve the purpose of anticipatory, curative, and distinctive clinical arenas, it requires meticulous metrics and statistical protocol to quantify capricious alternatives, notably within-subject and between-subject deviations [1]. Molecular-level occurrences—being dictated, scrutinized, and/or acknowledged by the sophisticated hordes of molecules or pairs of molecules—have a consequence on all biological aspects [2]. Evolutionary theories govern the assembly and evolution of biology. Lifeforms' layouts are evolved by the process of adaptation in order to employ their geometrical form, execute activities, and retain assistance [3]. The acronym for “disease” is recognized by ailments and manifestations that diverge from the acceptable phenotype, materializing biological pathways at the clinical level [4].

A primitive trait evident at all forms of biological hierarchy, through molecular associations to an extensive anatomical framework, is a biological asymmetry of phenotype. Compared to asymmetric behaviors, symmetry and complexity in biological organisms are more facile to encode and more likely to evolve during arbitrary mutations. Regardless, there is evolutionary pressure to induce asymmetry in many biological systems with high complexity, while symmetry might appear more frequently in biological components with less complexity. Maintaining the potential expertise of organ structure, asymmetry is essential to biological activity. In particular, anatomical left-right asymmetries exist in the human brain and are exhibited by all significant species of vertebrates, including the nervous systems of insects and invertebrates [5]. This asymmetry is frequently closely controlled in health, but variations beyond typical asymmetry constitute important diagnostic indicators in diseased circumstances. This natural asymmetry is frequently upset by illness, resulting in notable changes to morphology and physiology. For instance, asymmetric motor dysfunction, in which a particular region of the body appears more affected than the other region, is a frequent manifestation of neurodegenerative diseases such as Parkinson's disease [6]. Subclinical asymmetries in ocular framework and physiology are endorsed as prospective biomarkers for the prompt diagnosis of ophthalmic disorders. Researchers found that people with normal-tension glaucoma often exhibit interocular asymmetry in retinal nerve fiber layer thickness and intraocular pressure. The observation that a minor surge in intraocular pressure corresponds with lower retinal nerve fiber layer thickness underlines the clinical importance of evaluating interocular asymmetry. Comprehensive analysis of retinal nerve fiber layer thickness asymmetry alongside intraocular pressure discrepancies is essential for preliminary detection and prevention [7, 8]. Instances such as keratoconus and amblyopia exhibit contrasting asymmetrical layouts in corneal morphology and visual acuity, accordingly. The index of vertical asymmetry (IVA) is believed to be a very useful index for keratoconus assessment, stressing its importance of measuring asymmetrical corneal patterns for early recognition [9].

In the general populace, keratoconus strikes about 0.05% of people, which is mostly bilateral, noninflammatory, and asymmetric corneal ectasia [10–12]. This condition is denoted by a piecemeal alteration in the curvature of the cornea that causes the corneal surface to thin and protrude anteriorly. At the time the condition advances, surgical intervention is well suited to rectify the distortion of the visual stimulus that reaches the retina caused by this morphological corneal alteration [12]. Although ectasia is more frequently an untrammeled condition with a puzzling etiology and pathophysiology, there have been reports of a correlation between keratoconus and contact lens wearing, scratching eyes, Down syndrome, Leber congenital amaurosis, atopic illnesses, connective tissue diseases, tapetoretinal degeneration, inheritance, and mitral valve prolapse [10, 13]. Despite exhibiting good best corrected visual acuity (spectacle corrected), significant corneal distortion has been observed in keratoconus [14]. Studies also demonstrate and quantify corneal asymmetry in patients at the point of initial diagnosis of keratoconus [11]. These results implicitly indicate that the early phases of keratoconus can be effectively detected and described by corneal surface analysis methods [14]. Regarding the clinical management of astigmatism, which is one of the most common vision disorders, bilateral mirror symmetry and isorule consideration are important. Interference with the isorule pattern in astigmatism may arise with the presence of keratoconus as well [15].

While grading keratoconus, corneal thickness is frequently recognized as a clinically applicable pachymetric biomarker. Aberrant corneal thinning is a convenient identification in keratoconus eyes [16]. Average corneal dioptric strength is employed as an index to substantially reveal the clinical level of keratoconus. Greater values signify a deteriorating and clinical corneal ectasia. How the contour of the cornea varies from the central point to the peripheral margin is illustrated by the asphericity coefficient. This central and paracentral asphericity with a greater curve is evidence of keratoconus [17–20].

Arguably, comprehension of grasping the emergence of diseases and their therapy has been revolutionized by the intriguing evaluation of the interaction across biology, medicine, and creative endeavors; deep-seated illnesses remain difficult to define. This study endeavors to facilitate the mechanism that evokes the initial preclinical urge through a driving ocular assessment of the temporal dynamics between subjective sensation and measurable clinical criteria [21]. The efficacy of keratoconus detection at the refractive level as an efficient and reliable way is evaluated in this study.

## 2. Methodology

This is a prospective observational study with a single-arm case series design; no control group or intervention was employed. This study, conducted at Jupiter Eye Care, Bankura, from March 2024 to February 2025, aimed to investigate refractive symmetry deviation in a cohort of individuals aged 18–50 years, on 190 eyes, from 95 individuals. This study examines the relationship between refractive symmetry patterns and keratoconus severity within a single disease group, analyzing parameter deviations across varying severity levels. Stringent inclusion and exclusion criteria were implemented to ensure the homogeneity of the study population. Participants of both sexes, diagnosed with bilateral keratoconus (slit-lamp evaluation, followed by topographic detection) and bilateral astigmatism (more than or equal to 3.00 D), were considered in our investigation. Conversely, the research will not include participants with systemic conditions such as diabetes, hypertension, with a history of corneal injury, any ocular interventions including refractive surgeries, or those who have not gone 18 days without wearing contact lenses (soft and/or rigid lenses).

All participants underwent a comprehensive ophthalmic examination in the morning OPD (in order to avoid diurnal fluctuation and to maintain corneal biomechanics potentially stable), including general and ophthalmic history taking, visual acuity assessment using a standardized controlled lighted Snellen's chart and/or illuminated LogMAR chart, objective refraction using an auto-refractometer and the Heine Beta 200 retinoscope (HEINE, Germany), and subjective refinement considering sphere, cylinder, and axis notations. The Pentacam (OCULUS Optikgerate GmbH, Germany) evaluated the thickness of the thinnest corneal zone, average corneal diopter, and asphericity coefficient, which are essential and influential elements that have been considered to have a substantial impact on the progression of keratoconus. Collectively, such traits allowed a thorough in-depth assessment of the variables affecting refractive pattern in the course of keratoconus. Before evaluating keratoconus, all diagnostic instruments were calibrated according to the manufacturer's manual.

The bilateral symmetric deviations were quantified, revealing the refractive notations along the axes of cylindrical refractive elements of the yoke eye. Specifically, employing the principle of mirror symmetry (also known as mirror octant or enantiomorphism), bilateral symmetric deviation has been monitored. The ideal alignment is represented by a mirror octant or enantiomorphism, which is shown by a zero numerical variance among pairing axes. To identify different levels of enantiomorphism, the analysis goes beyond precise symmetry and employs graded levels (Table 1).

In Table 1, the maximum symmetric divergence is shown by Grade 5, whereas Grade 1 defines a perfect symmetric association. This approach enables a more enhanced comprehension of refractive interaction by embracing subtle bilateral correlations. Grading symmetry is a reliable strategy for exploring and efficiently resolving astigmatic refractive elements since it takes into account intrinsic morphological diversity [22]. Finally, once the symmetric deviations are identified, a conceptual framework for keratoconus detection was outlined to determine how the keratoconus markers—such as the thickness of the thinnest corneal zone, average corneal diopter, and asphericity coefficient—change in response to bilateral symmetric deviations.

A descriptive statistical approach provided a concise outlook of the data, while the Multiple *R* appraised the model fit. One-way ANOVA, guided by F-statistics, was solicited to analyze group variability, confirming robust speculation deriving out of the data set. A scatter plot was exploited to forecast the direction of mirror octant association of the variables. SPSS 29.0 software was utilized to perform the statistical analysis. A *p* value of less than 0.05 was considered statistically significant. According to our study hypothesis, all the factors with varying degrees of impact could greatly enhance a comprehensive diagnosis at a very early and clinical stage, allowing for prompt intervention with better patient outcomes. The study protocol received ethical approval from the Institutional Ethics Committee, NSHM Knowledge Campus, Kolkata (Ref. No: NSHMKOL/IEC/3/2024/PR-20), ensuring adherence to the principles outlined in the declaration. Before participation, informed consent was obtained from all subjects.

## 3. Results

### 3.1. Descriptive Statistics

As shown in Table 2, there existed no missing cases within the 52 female and 43 male participants, implying 100% data validity for either gender.

Data presented in Table 3 indicate that participants in the study range in age between 18 and 46 years. Considering a standard deviation of 8.39 and an average age of 29.31 years, the population exhibits moderate variability. All 95 respondents' data are comprehensive and void of incomplete quantities.

**Figure 1:**
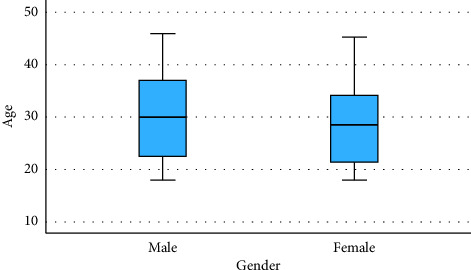
Boxplot presentation of study participants between age and gender. Source: Researchers' compilation, data were statistically evaluated using SPSS 29.0.

Apparently, as shown in Figure 1, there are no outliers in either of the male or female populations, pursuant to the boxplot contrasting of the age prevalence by gender, as no single statistical value is outside the whiskers. Concerning the females, males have a comparatively greater overall spectrum and an increased interquartile range (IQR), signifying a greater age distribution. As compared to the female cohort, whose ages have been more precisely centered toward the median, the male group's age variability tends to be greater.

**Figure 2:**
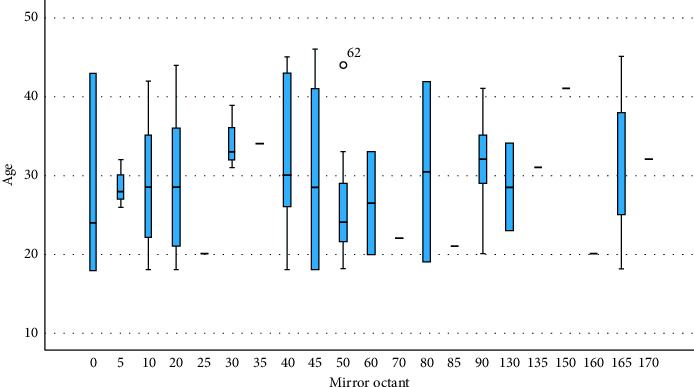
Boxplot presentation of study participants between age and mirror octant. Source: Researchers' compilation, data were statistically evaluated using SPSS 29.0.

Figure 2 depicts the dispersion of age across distinctive mirror octant angles which appear in the boxplot, which exhibits significant variance in overall central tendency and spread. Whereas specific octants, such as 35° and 135°, indicate consistently defined groups by age, certain others, including 0°, 40°, and 70°, display a broader IQR, implying higher age variability. There are outliers in several octants, most notably at 45°, which may indicate the presence of individuals with significantly higher ages. Subsequently, certain octants exhibit just median contours or minimal box dimensions, which suggest small samples. Mirror octant appears across an array of age categories, compared to the boxplot, implying that they do not appear age-driven. Every octant has a distinctive age pattern; however, certain ones, comprising 0°, 40°, and 70°, exhibit a wider age spectrum, despite certain others, such as 35° and 135°, possessing a considerably more restrictive range. Such a difference indicates that certain octants are frequently prevalent in particular age categories, despite all other mirror octants being distributed across an extent of ages. The results presented are preliminary, albeit, and a large number of participants are required to validate potential age-specific behavior and to ensure that the interpretations are accurate and relevant to an expanded population.

### 3.2. Regression Statistics

Multiple *R* (0.596434117) indicates a moderate positive correlation between the predictors (independent variables) and the response variable.

The mirror octant notion appears to possess considerable potential for identifying prospective keratoconus, as evidenced by the Multiple *R* statistic that indicates a moderately significant association across the model prediction and the data. This correlation, admittedly marginal, accentuates its biological relevance as an independent detection strategy.

According to Table 4, mirror octant (grade) is the only statistically significant predictor in the model. It has a strong positive effect on the response variable. Average corneal diopter in the left eye and thinnest corneal thickness in the left eye have borderline significance, suggesting that higher corneal dioptric value in the left eye and thinner corneas might influence the response variable.

## 4. ANOVA

Table 5 summarizes the key findings related to thickness, asphericity coefficient, thinnest corneal thickness, and mirror octant. The F-statistic is significant (*p* < 0.05), indicating that the regression model as a whole is statistically significant. At least one of the predictors contributes to explaining the variability in the response variable. The one-way ANOVA statistic (*F* = 6.86, *p* < 0.001) exhibits considerable deviation in average corneal intercept metrics, stressing the relevance of uncovering and reducing outliers that could impact the precision of the model. A systematically executed modified regression framework may enhance accuracy through reduction of distortion; however, extensive adaptation could disregard significant physiological alterations essential for the early recognition of keratoconus.

The cross (X) and horizontal line (—) represent the mean and median, respectively.

**Figure 3:**
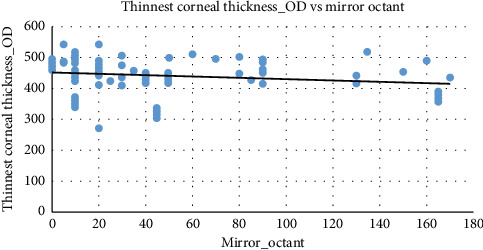
The scatter-plot between the thinnest corneal thickness of the right eye and mirror octant. Source: Researchers' compilation, data were statistically evaluated using SPSS 29.0.

The scatter-plot representation in Figure 3 illustrates a negative correlation between mirror octant and the thinnest corneal thickness of the right eye. As mirror octant increases, corneal thickness values tend to decrease slightly, indicating that mirror octant may capture some aspects of corneal structural integrity.

**Figure 4:**
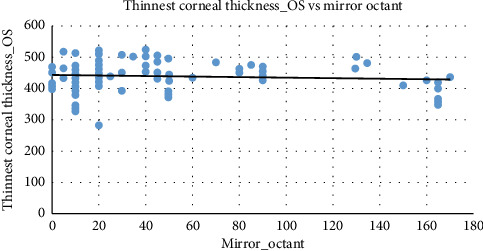
The scatter-plot between the thinnest corneal thickness of the left eye and mirror octant. Source: Researchers' compilation, data were statistically evaluated using SPSS 29.0.

The scatter-plot shown in Figure 4 presents the trend of a slight negative correlation between mirror octant and the thinnest corneal thickness of the left eye. As mirror octant increases, corneal thickness values tend to decrease slightly, consistent with the trend seen in the right eye. The clustering of data points around specific mirror octant values suggests that these classifications are prevalent across a range of corneal thicknesses.

Both right eye and left eye corneal thicknesses exhibit a negative relationship with mirror octant, where thinner corneas are associated with higher mirror octant classifications. The trends in right eye corneal thickness and left eye corneal thickness are nearly identical, highlighting bilateral consistency in corneal thickness and its influence on mirror octant classifications. This symmetry reinforces the reliability of using mirror octant to assess corneal characteristics across both eyes.

**Figure 5:**
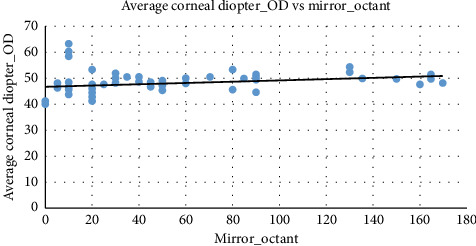
The scatter-plot between the average corneal diopter in the right eye and mirror octant. Source: Researchers' compilation, data were statistically evaluated using SPSS 29.0.

Figure 5 demonstrates the pattern of the scatter-plot with a very slight positive correlation between mirror octant and average corneal diopter in the right eye. As the mirror octant increases, the corneal diopter values tend to increase slightly, as indicated by the upward trend line.

**Figure 6:**
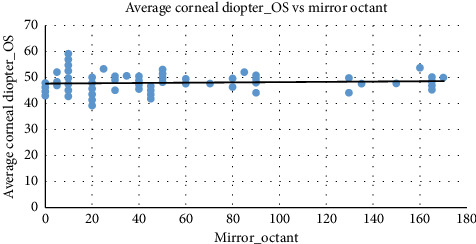
The scatter-plot between the average corneal diopter in the left eye and mirror octant. Source: Researchers' compilation, data were statistically evaluated using SPSS 29.0.

Similarly, as depicted in Figure 6, the average corneal diopter in the left eye and mirror octant exhibits a slight positive correlation. As mirror octant increases, corneal diopter values tend to rise marginally.

**Figure 7:**
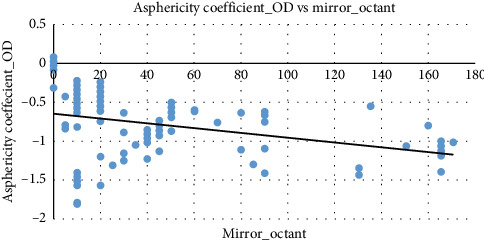
The scatter-plot between the asphericity coefficient of the right eye and mirror octant. Source: Researchers' compilation, data were statistically evaluated using SPSS 29.0.

Figure 7 provides a visual summary of the asphericity coefficient of the right eye demonstrates a negative correlation with mirror octant. As mirror octant increases, the asphericity coefficient of the right eye values tend to decrease (become more negative), as indicated by the downward-sloping trend line.

**Figure 8:**
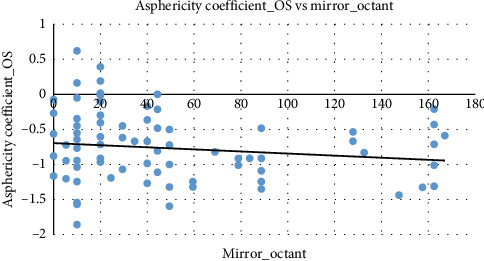
The scatter-plot between the asphericity coefficient of the left eye and mirror octant. Source: Researchers' compilation, data were statistically evaluated using SPSS 29.0.

Similarly, Figure 8 exhibits a slight negative correlation between mirror octant and asphericity coefficient of the left eye. As mirror octant increases, the asphericity coefficient of the left eye values tend to decrease slightly (become more negative), showing a pattern consistent with the asphericity coefficient of the right eye.

## 5. Discussion

In accordance with prior studies, as modest enantiomorphic axis aberrations frequently appear before apparent corneal curvature alteration, mirror octant symmetry is an effective template for prospective keratoconus identification. By improving sensitivity toward recognizing asymmetric corneal deviations, this type of investigation reduces the prospect of advancing earlier than significant topographic differences manifest and facilitates immediate intervention. Enantiomorphic axis aberrations and bilateral spatial aberration, which are frequently observed before evident topographic inclination, can potentially be sensitively identified by implementing mirror octant quantification. Identification of mirror-symmetry instances contributes to discerning subclinical symptoms, pursuing corneal scanning, and facilitating interventions such as cross-linking or frequent monitoring, considering early keratoconus manifests subtle, asymmetric meridional alterations opposed to identical meridian alterations. It enables preliminary and symmetrical inspection, enhances clinical sensitivity for asymmetric physiological changes, and governs the review of refractive surgical susceptibility [22]. To validate the usage and clinical utility of our novel keratoconus predictor by contrasting it with the existing metrics to ensure it is relevant. The study ascertains how the novel element mirror octant contributes as a nuanced outlook for the structural integrity of the cornea and envisions the severity of the keratoconus stages relevant to other established biomarkers. The adjacent clinical link with asphericity coefficient and corneal thickness, mirror octant is capable enough to speculating the refractive profile of the eye, which indicates the status quo of keratoconus. Corneal thickness decreases slightly with increasing mirror octant classifications, indicating that mirror octant may capture some aspects of corneal structural integrity. Thinner corneas with higher mirror octant classifications may require further clinical evaluation, especially for conditions such as progressive keratoconus or postsurgical outcomes. Mirror octant classifications are only minimally influenced by the average corneal diopter. The clustering of data points and the presence of outliers suggest the need for subgroup analyses or further investigation into clinical factors affecting mirror octant. Outliers with high mirror octant values at moderate average corneal diopter in the right eye level may represent unique clinical cases or specific subgroups in the population. The trends for the asphericity coefficient of the right eye and the asphericity coefficient of the left eye are similar, suggesting symmetry in corneal profiles between the left and right eyes. Both the values exhibit a negative relationship with mirror octant, where a more negative asphericity coefficient corresponds to higher mirror octant classifications. This consistent trend reinforces the role of corneal asphericity in determining mirror octant grades. Such symmetry aligns with clinical expectations for most individuals, though deviations might signal conditions affecting one eye more than the other. This information could be valuable for diagnosing and managing corneal disorders or planning refractive procedures.

Based on these results, mirror octant has good potential as a monitoring indicator and can upgrade diagnostic precision making in addition to reflecting the refractive profile continuum. Consequently, including mirror octant to keratoconus diagnostic protocol may greatly enhance the disease identification, clinical knowledge of corneal-refractive context, which fabricates one refractive diagnostic compel and tailors refractive analysis.

Nevertheless, encouraging study outcomes, this study has a number of imperfections. The model explains about 35.6% of the variability, which is moderate but indicates room for improvement. Additional predictors or nonlinear effects may enhance the model. The regression model is statistically significant all around, as indicated by the substantial F-statistic (*p* < 0.05). Particularly, contrasting with conventional keratoconus prognostic methods, in which multivariate regression predicted primarily 20.5% (*K*_max_) and 29.4% (LogMAR), a variation of 35.6% can be considered physiologically pertinent [23]. In view of biological variance, variability in measurement, and pathological heterogeneity, reasonable *R*2 values are reasonable in ocular trials. Subsequently, 35.6% model variability is reliable in early keratoconus assessment. In conclusion, mirror octant's applicability may be inadequate due to its subservience to corneal contour analysis, which, although obliging, may abstruse other salient clinical considerations. The subsistence of outliers advocates that mirror octant's predictive magnitude may fluctuate, which calls for validation employing extensive sample sizes. To corroborate that mirror octant is used effectively in the diagnosis and monitoring of refractive deviation in corneal ectasia, researchers and clinicians must work cooperatively to set a standardized quality for its use. A preprint version of this study has already been published for a better understanding of mirror octant [24].

## Figures and Tables

**Table 1:** The symmetric relationship and grade in terms of deviation [22].

Grade	Level of symmetric deviation (in degree)
1	0°
2	1°–5°
3	6°–10°
4	11°–15°
5	> 15°

**Table 2:** Descriptive statistics of study participants by gender and data completeness.

	Gender	Cases					
		Valid		Missing		Total	
		*N*	Percent (%)	*N*	Percent (%)	*N*	Percent (%)
Age	Male	43	100.0	0	0.0	43	100.0
	Female	52	100.0	0	0.0	52	100.0

*Note:* Source: Researchers' compilation, data were statistically evaluated using SPSS 29.0.

**Table 3:** Descriptive statistics of age of study participants.

	*N*	Minimum	Maximum	Mean	Std. deviation
Age	95	18	46	29.31	8.386
Valid *N* (listwise)	95				

*Note:* Source: Researchers' compilation, data were statistically evaluated using SPSS 29.0.

**Table 4:** Descriptive statistics for average corneal thickness, asphericity coefficient, thinnest corneal thickness, and mirror octant.

	Coefficients	Standard error	*t* stat	*p* value
Intercept	157.8328816	134.5244	1.173265861	0.243891387
Average corneal Diopter_OD^†^	−0.650225897	2.256025	−0.28821752	0.773865767
Average corneal Diopter_OS^‡^	−3.285803089	1.933306	−1.699577669	0.092783348
Asphericity Coefficient_OD	−44.81381124	28.39011	−1.578500744	0.118079876
Asphericity Coefficient_OS	−13.8864113	14.34115	−0.96829123	0.335583106
Thinnest corneal Thickness_OS	−0.237279409	0.124135	−1.911464624	0.059237005
Thinnest corneal Thickness_OD	0.139963286	0.11066	1.264805395	0.209318641
Mirror octant (Grade wise)	17.38543569	3.904504	4.452661613	2.50587E − 05

*Note:* Source: Researchers' compilation, data were statistically evaluated using SPSS 29.0.

^†^OD: Oculus Dexter, referring to the right eye, in Latin.

‡OS: Oculus Sinister, referring to the left eye, in Latin.

**Table 5:** One-way ANOVA results for interaction between average corneal.

**Intercept**	

F-statistic	6.86
Significance F	1.71E − 06

*Note:* Source: Researchers' compilation, data were statistically evaluated using SPSS 29.0.

## Data Availability

The data supporting this work are available from the corresponding author upon reasonable request.
